# Nitrogen addition and clonal integration alleviate water stress of dependent ramets of *Indocalamus decorus* under heterogeneous soil water environment

**DOI:** 10.1038/srep44524

**Published:** 2017-03-15

**Authors:** Zi-Wu Guo, Jun-Jing Hu, Shuang-Lin Chen, Ying-Chun Li, Qing-Ping Yang, Han-Jiang Cai

**Affiliations:** 1 Research Institute of Subtropical Forestry, Chinese Academy of Forestry, Fuyang, 311400, China; 2 Hangzhou Academy of Forestry, Hangzhou, 310016, China; 3 China National Bamboo Research Center, Hangzhou, 310012, China

## Abstract

Water and nitrogen are two of the most important factors for plant growth and development. However, little is known about effects of N on water translocation between connected bamboo ramets. We performed experiment connected *Indocalamus decorus* ramets in adjacent pots with different soil water contents and three N levels. We determined antioxidase activities, concentration of osmotic adjustment products, O_2_·^−^, MDA and photosynthetic pigments, and electrolyte leakage rate in paired unit. When N supply to supporting ramets increased, their electrolyte leakage rates and contents of O_2_·^−^ and MDA significantly increased, while antioxidase activities and contents of osmotic adjustment products and photosynthetic pigments in connected dependent ramets increased markedly as their electrolyte leakage rates and contents of O_2_·^−^ and MDA decreased greatly. When N addition to dependent ramets increased, antioxidant enzyme activity and contents of osmotic adjustment products and photosynthetic pigments decreased in both ramets, but electrolyte leakage rates and O_2_·^−^ and MDA contents increased significantly. Therefore, N addition to either supporting or dependent ramets can improve water integration among *I. decorus* ramets. N addition to supporting ramets promotes water translocation and alleviates water stress of dependent ramets, but N addition to dependent ramets exacerbates drought stress damage to dependent ramets.

Clonal plants produce new modules with uniform genetic features that are potentially physiologically independent^1^. These modules (the ramets) are formed by vegetative propagation and stay connected to the parent organs at least until they are established, thus forming sets of connected ramets, or clonal fragments^1^,^2^,^3^. Clonal plant responses to heterogeneous environments are complex because they have modular construction and are able to grow laterally into new exploitable space, while at the same time retaining physiological connectivity^2^. Ramets connected by stolons or rhizomes not only expand into new growing space for resource acquisition but also share water, carbohydrates, and nutrients among the connected units through clonal integration^3^,^4^. Clonal integration, which is the movement of resources, signals, or pathogens among connected ramets within clonal fragments, permits connected units to access different levels of resource availability and reduces competition among the modules^5^,^6^,^7^. Many studies have shown that clonal integration facilitates the establishment of newly produced ramets, improves survival and growth in stressful environments^8^, and helps genets occupy open space^5^,^6^,^7^,^8^. However, little is known about physiological integration of bamboo species in heterogeneous environments^9^,^10^,^11^,^12^,^13^, although many studies have been made on herbaceous plants such as *Fragaria orientalis*^14^, *Alternanthera philoxeroides*^15^, *Potentilla anserina*^16^, and *Zoysia japonica*^17^.

Patchy fertilization promoted shoot production of the bamboo *Phyllostachys pubescens* in the unfertilized plots compared to uniformly fertilized patches as a result of clonal integration^11^. Under heterogeneous resource conditions, the translocation of nitrogen and assimilates among ramets of the bamboo *Sasa palmata* was greater than that in homogeneous conditions^12^,^13^. Furthermore, the resource contrast and ratios of supporting ramets to dependent ramets impacted the translocation efficiency of the dwarf bamboo *Indocalamus decorus*, and increasing water contrast and the ratios enhanced water integration strength^9^,^10^.

Water and nitrogen (N) are important constraints on plant survival, growth, and development. As the global climate changes, shifts in precipitation patterns and enhanced N deposition have exacerbated the spatial and temporal heterogeneity of water and nitrogen distribution^18^,^19^. Drought is now among the most destructive catastrophic climate elements. Seasonal or periodic droughts are frequent, even in areas with abundant rainfall^20^. China has recently become one of three areas on the planet with the highest rates of N deposition^21^,^22^. Increased N input may exert strong effects on both the structure^23^,^24^ and function^25^,^26^ of terrestrial ecosystems. N deposition may inhibit plant growth by disrupting the balance of elements, reduce biological diversity by soil acidification, and cause forest degeneration under the most serious circumstances^27^. Because changing N availability may greatly affect the growth rate of individual ramets, and may also influence water absorption and utilisation, it may further alter the potential source-sink relationship of connected ramets and thus affect their water integration. However, few studies have tested the effect of soil N availability on water translocation among connected ramets, especially for bamboo species.

Bamboo is widely distributed in Asia, Africa, and Latin America, where it is among the most important non-timber tropical forest products. More than 1250 bamboo species in 75 genera have been reported worldwide^28^. China’s bamboo species account for more than 40% of the world total. Bamboo is a clonal plant with ramets connected by rhizomes. It grows rapidly, can be sustainably managed after reafforestation, and provides a variety of useful products and services for human beings. It plays important roles in regional water and soil conservation efforts, carbon sequestration, oxygen emission, and climate regulation, making it an important element of terrestrial ecosystems. Many studies have reported the physiological and ecological responses of bamboo to water stress, but most have focused on individual plants^29^,^30^,^31^ or forest stands^32^,^33^ and little is know about the ecological mechanisms and effects of water translocation among bamboo ramets connected through rhizomes^9^,^10^,^11^,^12^,^13^.

We conducted a glasshouse experiment to investigate changes in oxidative stress levels, antioxidant activity, and chlorophyll content of *Indocalamus decorus* supplied with different N levels under varied soil water potentials. The following questions were addressed: (1) Are the antioxidant activities and chlorophyll contents of supporting ramets growing in well-watered soils affected by the integration of dependent ramets subjected to water deficiency, i.e. does water transport among ramets occur at a threshold on the water supply gradient? (2) Does N addition affect clonal integration among the ramets subjected to different water supply? (3) Does N addition exacerbate or alleviate the injury inflicted by the drought stress experienced by dependent ramets? We predicted that physiological integration moderates the negative effect of drought stress on dependent ramets and that N addition benefits water integration among ramets.

## Results

Repeated-measures ANOVA showed that both N addition and the duration of the treatment had significant effects on leaf antioxidase activities (SOD, POD and CAT), leaf MDA and O_2_·^−^ contents, leaf photosynthetic pigment contents (Chlorophyll a, Chlorophyll b and Carotenoid), and leaf osmotic-adjustment product contents (soluble protein and total soluble sugar) of ramets; the interaction term for these two effects was also significant (Table 1).

### Leaf antioxidase activity responses to N addition in ramets growing at high and low soil water potentials

**Figure 1:**
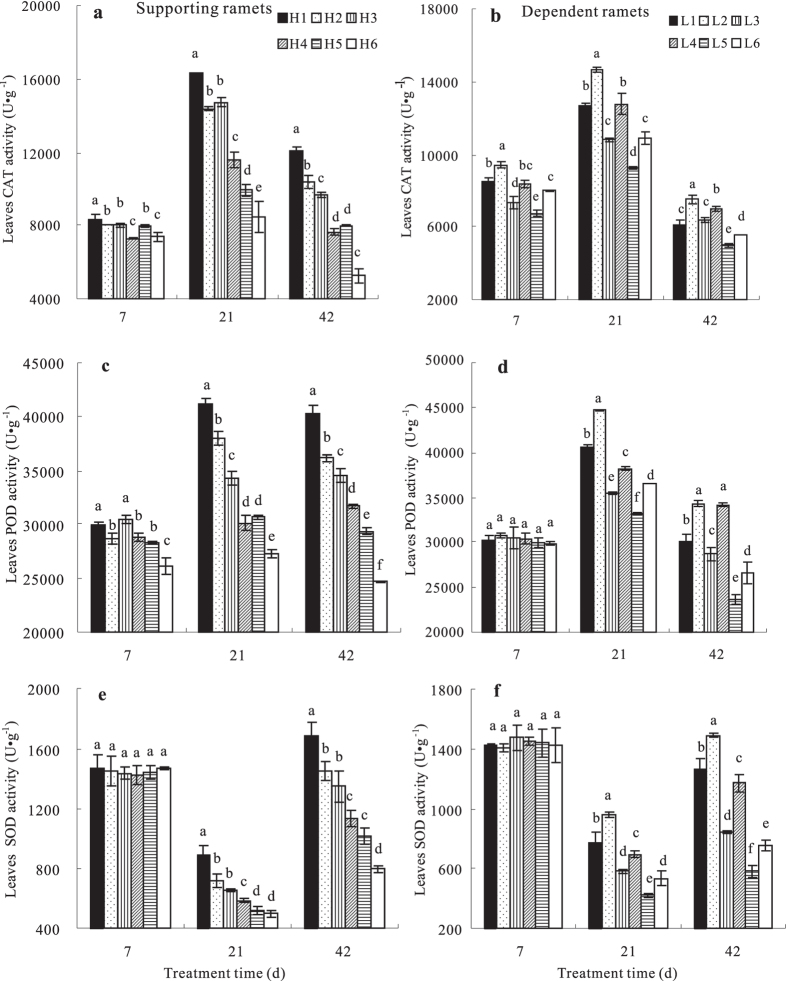
Leaf antioxidant enzyme activities of *Indocalamus decorus* ramets subjected to different experimental treatments (units of enzyme activity are U g^−1^ FW). *H1 and L1 refer to supporting ramets and dependent ramets in the 0N:1N treatment, respectively; H2 and L2 refer to supporting ramets and dependent ramets in the 0N:2N treatment, respectively; H3 and L3 refer to supporting ramets and dependent ramets in the 1N:0N treatment, respectively; H4 and L4 refer to supporting ramets and dependent ramets in the 1N:2N treatment, respectively; H5 and L5 refer to supporting ramets and dependent ramets in the 2N:0N treatment, respectively; H6 and L6 refer to supporting ramets and dependent ramets in the 2N:1N treatment, respectively. The pots were subjected to three levels of N fertilisation: 0N (no N addition), 1N (15 g urea pot^−1^), and 2N level (30 g urea pot^−1^). The two pots in which each of the connected pairs of ramets were grown were subjected to a range of N treatments (pairwise: 0N:1N, 0N:2N, 1N:2N, 1N:0N, 2N:0N, and 2N:1N). Different letters in the bars indicate significant pairwise differences among different N addition treatments.

Across treatments, the CAT and POD activities of ramets first increased and then decreased gradually with time, and the SOD activity first decreased and then increased. N addition significantly affected leaf antioxidase activity (*p* < 0.05) in the paired pots (Fig. 1). With respect to N supply levels to the dependent ramets, the higher the N supply to the supporting ramets was, the lower their antioxidase activity became, indicating that water transport from the supporting ramets to the dependent ramets reduced scavenging activity on reactive oxygen species (ROS) in the supporting ramets. However, the antioxidase activity in the connected dependent ramets increased, indicating that water transport strengthened the ROS scavenging activity in the dependent ramets. In terms of N supply levels to the supporting ramets, the higher the N supply to the dependent ramets was, the lower the antioxidase activity in the connected supporting ramets became; however, the antioxidase activity of the dependent ramets increased. These results suggest that clonal integration alleviated the water stress in the dependent ramets and that N addition to the supporting ramets enhanced water integration. The higher the N supply to the supporting ramets was, the more the dependent ramets profited from water transport and the greater the cost for the supporting ramets became.

### Effects of N addition on leaf MDA and O_2_·^−^ contents and electrolyte leakage rate of ramets growing at high and low soil water potentials

**Figure 2:**
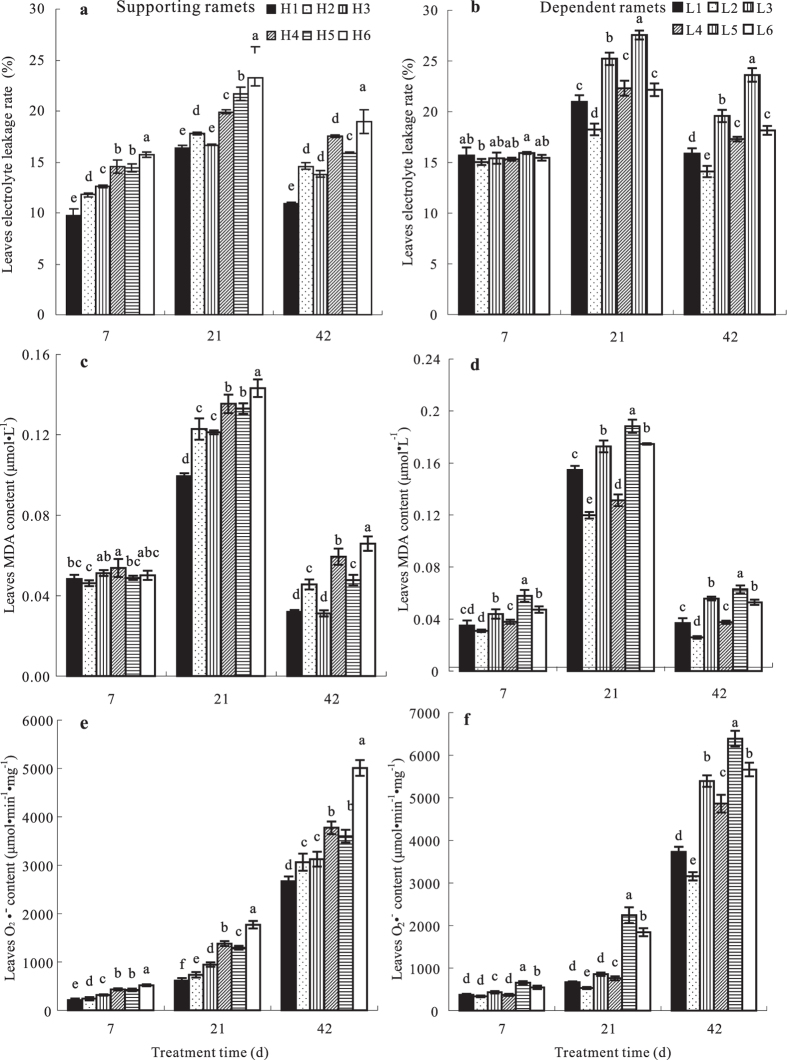
Leaf electrolyte leakage rate (%), contents of malondialdehyde (MDA, μmol l^−1^), and reactive oxygen species content (μmol min^−1^ mg^−1^) of *Indocalamus decorus* ramets subjected to different experimental treatments. See legend to Fig. 1 for an explanation of treatments.

As the duration of treatment increased, leaf O_2_·^−^ content increased significantly (*p* < 0.05); MDA contents and electrolyte leakage rate increased initially and decreased thereafter. Electrolyte leakage rates and contents of MDA and O_2_·^−^ in both types of ramets differed significantly (*p* < 0.05) among N addition treatment (Fig. 2). With respect to N supply levels to the dependent ramets, leaf electrolyte leakage rates and contents of MDA and O_2_·^−^ in the supporting ramets increased significantly as the N supply to them increased, but the values of these parameters decreased significantly in dependent ramets. The ROS contents of the dependent ramets decreased, and peroxidation of their cell membrane lipids fell when N was added to the supporting ramets, indicating that water transport between ramets was improved, with resulting benefits for the dependent ramets. As for N supply levels to the supporting ramets, the leaf electrolyte leakage rate and contents of MDA and O_2_·^−^ in both supporting and dependent ramets increased significantly with increasing N addition rates to the dependent ramets, indicating that N addition to dependent ramets exacerbated water stress and decreased clonal system fitness. Thus, N addition to both types of ramets increased their leaf O_2_·^−^ and MDA contents and elevated peroxidation of cell membrane lipids, indicating that water transport among the ramets was improved (with consequent increased costs for the supporting ramets).

### Effects of N addition on the leaf photosynthetic pigment contents of ramets growing at high and low soil water potentials

**Figure 3:**
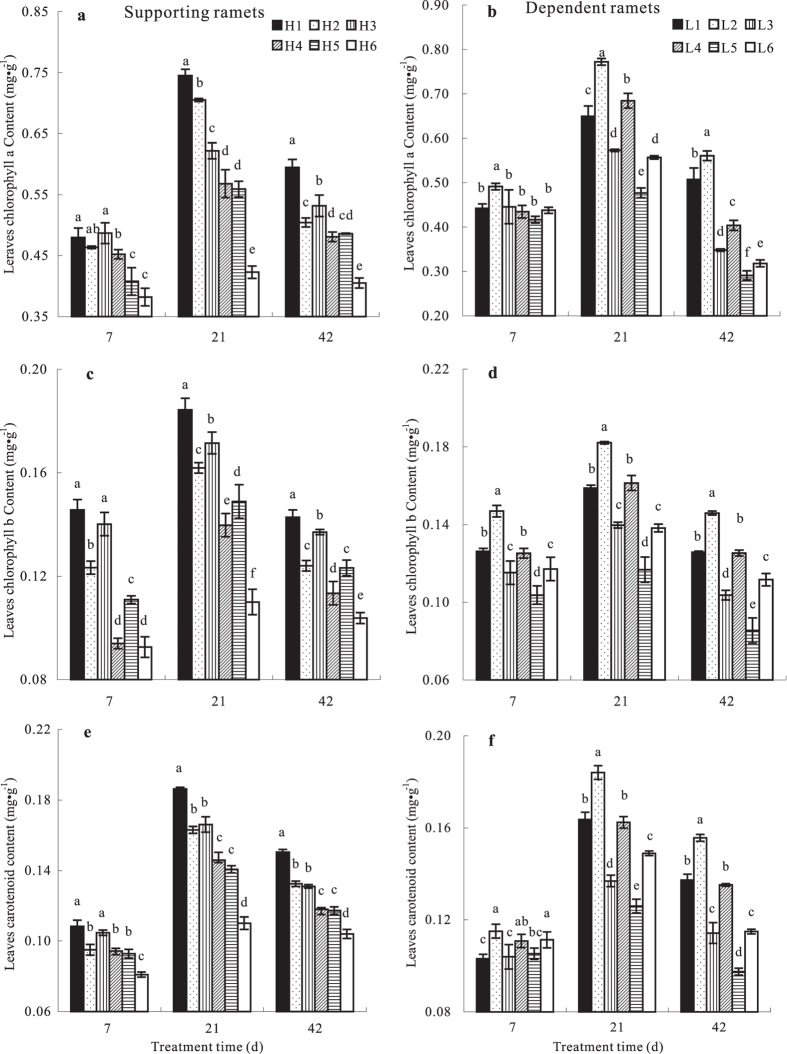
Leaf photosynthetic pigment contents (mg g^−1^) in *Indocalamus decorus* ramets subjected to different water potentials. See legend to Fig. 1 for an explanation of treatments.

As the treatment time increased, the leaf photosynthetic pigment contents of dependent and supporting ramets first increased and then declined. N addition significantly affected (*p* < 0.05) the leaf photosynthetic pigment contents of the connected ramets (Fig. 3). With regard to N supply levels to the dependent ramets, the leaf photosynthetic pigment contents of the supporting ramets decreased significantly as the N supply rate to them increased, indicating that water transport lowered the photosynthetic pigment contents of the supporting ramets, thereby reducing their photosynthetic capacity, but it significantly increased the pigment contents in the dependent ramets. Thus, alleviation of water stress through physiological integration inhibited the degradation of photosynthetic pigment in the dependent ramets, thereby enhancing their photosynthetic capacity. As for N supply levels to the supporting ramets, leaf photosynthetic pigment contents of both supporting and dependent ramets decreased significantly as N addition to the dependent ramets increased, indicating that N addition to the dependent ramets enhanced water transport, thereby greatly increasing the costs for the supporting ramets. Therefore, the profits conferred to the dependent ramets through physiological integration inadequately offset the costs of water loss for the supporting ramets. Furthermore, N addition to the dependent ramets exacerbated water stress for the dependent ramets; the fitness of the whole clonal system obviously decreased under these conditions.

### Effect of N addition on the leaf osmotic-adjustment product contents of ramets growing at high and low soil water potentials

**Figure 4:**
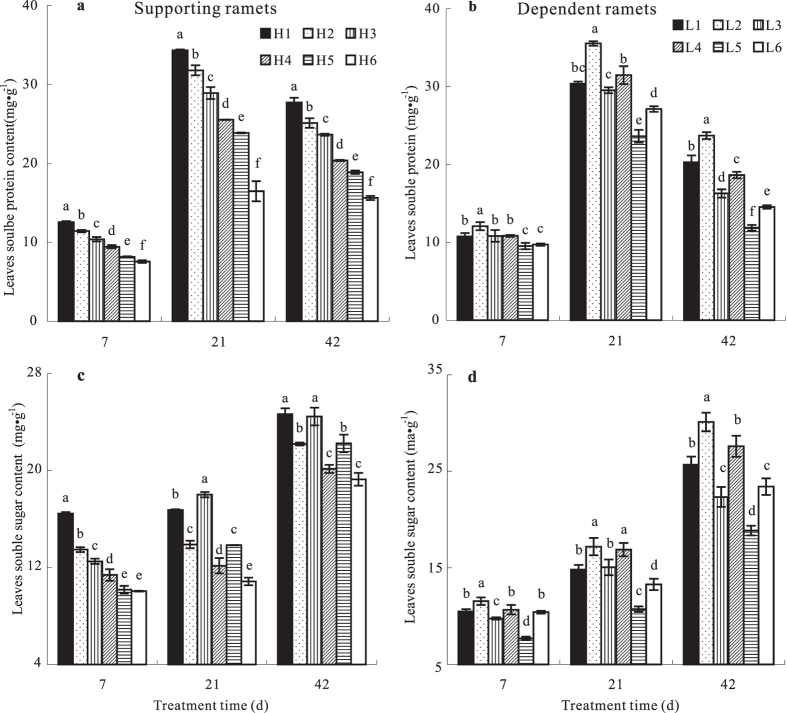
Leaf relative soluble protein and soluble sugar contents (mg g^−1^) in *Indocalamus decorus* ramets subjected to different experimental treatments. See legend to Fig. 1 for an explanation of treatments.

As the duration of treatment increased, the total soluble sugars in leaves increased significantly; the soluble protein content initially increased, but then decreased. There was a significant difference (*p* < 0.05) in the contents of total soluble sugars and soluble protein among the N addition treatments (Fig. 4). In terms of N supply levels to the dependent ramets, the contents of leaf total soluble sugars and soluble protein in the supporting ramets decreased significantly as N supply to them increased, indicating that the osmotic adjustment abilities of the supporting ramets had decreased due to water loss. However, these abilities increased significantly in the dependent ramets, indicating that the osmotic adjustment ability of dependent ramets had increased due to water acquisition through the integrated clonal system. With respect to N supply levels to the supporting ramets, the contents of leaf total soluble sugars and soluble proteins in both supporting and dependent ramets decreased significantly with increasing N addition to the dependent ramets, indicating that increasing the osmotic potential of the supporting ramets enhanced water transport to the dependent ramets, but at heavy cost for the supporting ramets.

## Discussion

Nitrogen (N) is an key element for plant growth and development. It strongly affects water absorption and utilisation, especially in arid environments. Previous studies have shown that increasing plant N content alleviates leaf dehydration under drought stress. When soils are well watered, N addition benefits plant growth and improves the regulation of plant water potential. Under moderate drought stress, N addition has no obvious effects on water potential, but under severe drought stress, N addition degrades soil water conditions, exacerbates drought stress on plants, and gradually reduces root water potential. Thus, N addition negatively impacts the regulation of plant water potential^34^. Our study detected an obvious decline in the osmotic adjustment ability of drought-stressed ramets (dependent ramets) as N supplementation levels increased under conditions of low water potential; at the same time, soil water conditions clearly deteriorated, and leaf water content and utilisation efficiency decreased markedly^35^. Leaf ROS in dependent ramets clearly increased as their antioxidase activity declined with increasing N supplementation. Thus, drought stress caused a decline in the oxygen scavenging ability and exacerbated the oxidative damage^36^. Furthermore, N addition to the dependent ramets made their photosynthetic pigments more susceptible to drought stress; the biosynthesis of these pigments was inhibited, and their degradation accelerated, resulting in strong declines in photosynthetic pigment content. The obvious increases in the electrolyte leakage rate and the contents of MDA and O_2_·^−^ also heavily damaged cell membrane permeability. In heterogeneous conditions, clonal connection can act as cooperative system, and the effects of stress on one ramet can be ameliorated by another connected ramet under benign conditions^37^,^38^,^39^. Many previous studies have demonstrated that the movement of resources among ramets mitigated the impacts of resource heterogeneity on clonal plants and enhanced their adaptability in stressful habitats^12^,^13^,^38^,^39^. In our experiment, when N addition to the dependent ramets was increased, the antioxidase activity in the connected supporting ramets, which acted as the water suppliers or water sources, decreased markedly. Furthermore, the contents of the photosynthetic pigments and osmotic adjustment products declined in the supporting ramets, while leaf MDA and O_2_·^−^ contents and electrolyte leakage rate increased, indicating that N addition to dependent ramets exacerbated damage caused by drought stress and promoted bulk water transport from the supporting ramets to sustain normal growth and development in ramets subjected to drought stress. Overall, the resource heterogeneity or contrast impacted the translocation among the ramets, the larger the contrast between water availability, the larger the amount of support the depending ramet received from the supporting one^38^. Therefore, the greater the water transport between ramets became, the higher were the costs borne by supporting ramets through the consumption of energy required for water integration. Hence, N addition to the dependent ramets promoted costly water transport from the supporting ramets and exacerbated stress damage in the dependent ramets when habitat conditions were heterogeneous.

Individual ramets in the same clonal system tend to specialise in the acquisition of the most abundant resources in their vicinity, and different ramets play complementary roles in resource-scattered environments^40^. Ramets of dwarf bamboos in shaded habitats can obtain assimilates from the ramets in open habitats, and transport nitrogen to those ramets in open habitats, then the fitness of whole clone enhanced^12^,^13^. Ramets respond not only to water status in their own habitats (local effect) but also to the water status in their connected ramet habitats (non-local effect). Local effects may be changed by the impacts of non-local effects generated by environmental stress and variable resource availability^41^. We found that the physiological plasticity of ramets subjected to different N addition treatments was clearly varied. We detected significant differences in physiological indices among ramets in different N addition treatments; water transported from supporting ramets to dependent ramets promoted the growth and development of dependent ramets in drought-stressed habitats. As N addition to supporting ramets was increased, their antioxidase activities and the contents of photosynthetic pigment and osmotic adjustment products clearly increased in concert; the contents of O_2_·^−^ and MDA and the electrolyte leakage rate decreased in the dependent ramets, but increased in the supporting ramets, which bore the increased costs of water supply to the dependent ramets and suffered consequent declines in adaptability and fitness. Environmental heterogeneity, resource availability and development stage of ramets are important influence factors of clonal integration. Normally, clonal plants in heterogeneous environments show a higher capacity for integration and division of labor. Source–sink relations caused by resource availability and its contrast are the main drivers of water integration of plants. However, under homogenous environment, clonal ramets also transport and share the resource due to different ability of resource absorption and utilisation. When water is sufficient in a habitat (patch), enhancement of soil N content promotes clonal growth and water absorption^42^. Water contrast of the two patches enhances and also promotes clonal integration. In our experiment, N addition and the water gradient between sources and sinks promoted water transport between ramets, to the benefit of the dependent units. Water integration necessarily results in costs and losses for the supporting ramets, inhibiting water transport outwards from these modules and reducing their growth^43^.

## Conclusions

Water transport from supporting ramets to dependent ramets benefited dependent ramets but reduced the supporting ramets’ water supply. N addition had obvious effects on water integration and on the costs/benefits balance. N addition to either the supporting or dependent ramets enhanced water physiological integration strength in the *I. decorus* clonal system to the benefit of the dependent ramets (which had improved access to water) and at a cost to the supporting ramets (which lost water). N addition had different effects on supporting and dependent ramets. N addition to the dependent ramets degraded their water supply environment and seriously damaged them, whereas N addition to the supporting ramets had positive effects on these modules by increasing their adaptability and fitness. Therefore, managers should add N nutrients to bamboo forests where water is abundant to promote water transport to connected stands in water deficient habitats, which would bring adaptability and fitness benefits to the whole bamboo forest. If N is to be added to bamboo forests in water-deficient habitats, the level of fertilisation must be controlled to avoid damage to plants.

## Methods and Materials

### Experimental site

The experimental site was located in an ornamental bamboo garden in Taihuyuan Township, Lin’an City, Zhejiang Province, China (29°56′–30°23′N, 118°51′–119°72′E). The region is located in a subtropical monsoon climatic zone, where conditions are warm and humid, and four distinct seasons are discernible. The annual rainfall is 1250–1600 mm, and the average annual temperature is 15.4 °C; the average January temperature is 3.2 °C, and the average July temperature is 29.9 °C. The lowest temperature recorded was −13.3 °C, and the highest, 40.2 °C. The annual average active cumulative temperature (≥10 °C) is 5100 °C, the average annual frost-free period is 235 days, and the cumulative annual daily sunshine is 1850–1950 hours.

### Experimental materials

The study bamboo (*Indocalamus decorus*) completes morphological development in the early growing season (before July) and its morphology will not change much in the rest of the growing season (because it has no secondary growth for lack of lateral meristem). We established clonal systems of *I. decorus* by rhizome propagation in a glasshouse during March 2013 before the shoot sprouts. We divided 144 plastic pots (40 × 40 × 30 cm: length × width × height) into 72 sections, and pairs of separated pots in each section were kept adjacent to one another in each of the treatments. The potting medium was a uniform mixture (3:1, vol:vol) of red soil and silver sand, with pH of 5.8, hydrolysable N concentration of 198.47 mg kg^−1^, available phosphorus concentration of 67.25 mg kg^−1^, and available potassium concentration of 74.16 mg kg^−1^. We selected 27 rhizomes (2 years old, 3 mm in diameter, and 50 cm in length) for clonal system establishment. For each clonal system, we arranged two pots in close alignment and fastened them securely to a panel. To allow rhizomes to pass between adjacent pots, we drilled a small hole of 0.5 cm in diameter in each pot. After planting the rhizomes, we sealed the holes with mud so that water and nutrients in the two adjacent pots was unlikely to interfere. We added soil medium to a depth immediately below the drilled holes, and threaded sections of rhizome through the holes so that 25-cm lengths remained in each pot. Soil medium was then added until it reached a level 5 cm below the pot rims. The experimental bamboo material was manually watered at regular intervals; bamboo shoots and weeds were removed as necessary. The number of ramets was controlled by removing small, fragile units. The numbers in each pot ranged from 10 to 12 after 5 months.

### Experiment design

At the beginning of the experiment in August 2013, we divided 36 pairs of pots containing 20 similarly sized ramets (10 ramets per pot) into six groups to be treated with different N supplements. Urea (N content 46%) was selected as the N source and was provided at three concentration levels: 0N (no N addition), 1N (15 g urea pot^−1^), and 2N (30 g urea pot^−1^). The two pots in each connected pair were maintained with different water contents: 90 ± 5% [high water potential] *vs*. 30 ± 5% [low water potential] of the field water holding capacity (i.e. 34% soil water content) due to its high intensity of water translocation among ramets in this water contrast^9^ and subjected to a range of N treatments (pairwise: 0N:1N, 0N:2N, 1N:2N, 1N:0N, 2N:0N, and 2N:1N) with the N content of the six treatment (pairwise: 0g N:6.9g N, 0g N:13.8g N, 6.9g N:13.8g N, 6.9g N:0g N, 13.8g N:0g N, and 13.8g N:6.9g N). N was added to each pot 6 hours after the water treatments were started, and it was applied only once during the experiment. Soil water content was determined twice a day at 7:00 AM and 18:00 PM by soil water determination meter (WET-2 Sensor), then soil water was supplemented according to the treatment design. The entire experimental configuration was deployed in a randomised complete block design. Within each pair of pots, the bamboo units in the pot with a high water potential were identified as supporting ramets, and those in the other pot as the dependent ramets. In the mornings (09:00–10:30) of treatment days 7, 21, and 42, we randomly selected a mixed sample of 10–12 mature leaves from each pot and determined their primary physiological and biochemical indices.

### Photosynthetic pigment content and electrolyte leakage rate

We measured chlorophyll contents following the procedures of Zhang & Chen^44^ with slight modifications. Fresh leaves (50 mg) were extracted in a mixture of 2.5 ml acetone and 2.5 ml ethanol for 24 h in darkness at room temperature. After extraction, we measured the absorbances spectrophotometrically at 447 nm, 663 nm, and 645 nm to calculate the contents of chlorophyll *a* and *b* and carotenoids.

The electrolyte leakage rate was measured as electric conductance. We submerged 28 8-mm discs in 20 ml distilled water, vacuum infiltrated them for 30 min, and then shook them for 2 h, after which we measured the initial electric conductance (S1). Samples were digested in water at 100 °C for 30 min, after which we determined the final electric conductance (S2). The electrolyte leakage rate was calculated from the following expression:


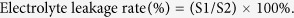


### Superoxide anion radical (O_2_·^−^) and MDA contentsh

O_2_·^−^ content was determined by the hydroxylamine oxidation method of Ke *et al*.^45^ with slight modifications. Fresh leaves (0.5 g) were ground in liquid nitrogen with 5 ml 50 mM (pH 7.8) phosphate buffer. The homogenate was centrifuged at 10,500 × *g* for 20 min at 4 °C. We added 1 ml of hydroxylammonium chloride (1 mM) to 0.5 ml of the supernatant and incubated the mixture for 10 min at 25 °C. We subsequently added 1 ml 4-aminobenzenesulfonic acid (17 mM) and 1 ml α-aphthylamine (7 mM) to the mixture and held it for 20 min at 25 °C to allow colour development. Specific absorption was measured at 530 nm. Sodium nitrite was used as the standard solution to calculate the content of O_2_·^−^.

The malondialdehyde (MDA) content was determined by the thiobarbituric acid (TBA) method. We transferred 1.5 ml of the supernatant to a capped test tube containing 2.5 ml of 0.5% TBA solution. The mixture was incubated in a boiling water bath for 20 min then centrifuged. We measured absorbances at 532 nm, 600 nm and 450 nm, and calculated MDA concentration as follows^46^:


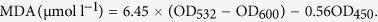


### Enzyme extraction and assay

Fresh leaves (0.5 g) were ground in liquid nitrogen. The ground samples were individually homogenised in 10 ml of 50 mM phosphate buffer (pH 7.8) in an ice bath. The homogenates were centrifuged at 10,500 × *g* for 15 min at 4 °C. The supernatant was used for the following enzyme assays.

Superoxide dismutase (SOD; EC 1.15.1.1) activity was assayed by measuring the inhibition of the photochemical reduction of nitroblue tetrazolium (NBT). The 3-ml reaction mixture contained 50 mM phosphate buffer (pH 7.8), 13 mM methionine (Met), 75 μM NBT, 2 μM riboflavin, 0.1 mM EDTA, and 0.05 ml of enzyme extract. Reaction mixtures were illuminated for 30 min under a photon flux density of 75 μmol m^−2^ s^−1^. One unit of SOD activity was defined as the amount of enzyme required to cause 50% inhibition of NBT at 560 nm^46^.

Peroxidase (POD; EC 1.11.1.7) activity was measured using the guaiacol oxidation procedure. The assay mixture contained 1 ml of 0.3% H_2_O_2_, 0.95 ml of 0.2% guaiacol, and 1 ml of 50 mM phosphate buffer (pH 7.0). We added 0.05 ml of enzyme solution to the reaction mixture to make up a total volume of 3.0 ml. To calculate POD activity, we began recording changes in the mixture absorbance at 470 nm 30 s after the reaction had started, and continued at 30-s intervals for a total of 3.0 min.

Catalase (CAT; EC 1.11.1.6) activity was determined by directly measuring the decomposition of H_2_O_2_ at 240 nm. The reaction mixture comprised 1.0 ml 0.3% H_2_O_2_, 1.9 ml H_2_O, and 0.1 ml enzyme solution. We mixed 0.1 ml enzyme solution with 2.9 ml reaction mixture, then added 1.0 ml 0.3% H_2_O_2_ to start the reaction. We recorded absorbances at 240 nm at 30-s intervals for 3.0 min. A 0.01 min^−1^ decrease in OD was defined as an activity unit^46^.

### Analysis of osmotic adjustment products

Total soluble sugar content was measured using the method of Quan *et al*.^47^ with slight modifications. Total soluble sugars were extracted with 80% ethanol and measured by the anthrone reaction at 630 nm using glucose as a standard.

The soluble protein content was estimated by the Coomassie Brilliant Blue G-250 dye-binding method^48^ with slight modifications. Fresh leaves (0.5 g) were homogenised in 5 ml of 50 mM phosphate buffer (pH 7.8). The homogenate was centrifuged at 10,000 × *g* for 10 min. We mixed 1 ml of supernatant with 5 ml Coomassie Brilliant Blue G-250 and read the absorbances of the mixture at 595 nm.

### Statistical analysis

Microsoft Excel 2007 was used for data sorting and constructing graphical plots. One-way ANOVA was used to test the effect of N addition on leaf physiological indices of supporting ramet and dependent ramats for each treatment time. We used repeated measures ANOVA to analyze the effect of N addition or treatment time, and their interaction on leaf physiological indices of supporting ramet and dependent ramats for all treatment time. SPSS 16.0 statistical software (SPSS, Inc., Chicago, IL, USA) was used to perform one-way analyses of variance (ANOVA) and repeated measures ANOVA; the least significant difference (LSD) multiple comparisons test was used for pairwise comparisons (*p* < 0.05). All values presented are means ± SD.

## Additional Information

**How to cite this article**: Guo, Z.-W. *et al*. Nitrogen addition and clonal integration alleviate water stress of dependent ramets of *Indocalamus decorus* under heterogeneous soil water environment. *Sci. Rep.* **7**, 44524; doi: 10.1038/srep44524 (2017).

**Publisher's note:** Springer Nature remains neutral with regard to jurisdictional claims in published maps and institutional affiliations.

## Figures and Tables

**Table 1:** Summary statistics of repeated-measures ANOVA on leaf physiological indices in *Indocalamus decorus* ramets subjected to different experimental treatments.

Ramets	Experimental indexes	N addition (N)		Treatment time (T)		N × T	
		*F5,* a	*P*	*F2,* b	*P*	*F10,* c	*P*
Supporting ramets	CAT activity	353.728	<0.001	1328.062	<0.001	77.112	<0.001
	POD activity	809.311	<0.001	472.057	<0.001	75.454	<0.001
	SOD activity	66.921	<0.001	876.207	<0.001	21.519	<0.001
	MDA content	83.459	<0.001	4409.362	<0.001	27.488	<0.001
	Electrolyte leakage rate	187.259	<0.001	1014.631	<0.001	13.263	<0.001
	O_2_·^−^ content	110.696	<0.001	4033.953	<0.001	31.821	<0.001
	Chlorophyll a content	251.972	<0.001	460.295	<0.001	25.560	<0.001
	Chlorophyll b content	184.859	<0.001	652.175	<0.001	17.630	<0.001
	Carotenoid content	480.746	<0.001	2295.335	<0.001	39.227	<0.001
	Soluble protein content	682.126	<0.001	7589.709	<0.001	98.848	<0.001
	Total soluble sugar content	310.632	<0.001	3084.207	<0.001	26.040	<0.001
Dependent ramets	CAT activity	449.792	<0.001	2173.230	<0.001	20.248	<0.001
	POD activity	136.000	<0.001	1567.656	<0.001	71.936	<0.001
	SOD activity	63.512	<0.001	611.218	<0.001	22.155	<0.001
	MDA content	203.585	<0.001	10364.242	<0.001	39.409	<0.001
	Electrolyte leakage rate	94.621	<0.001	457.825	<0.001	17.897	<0.001
	O_2_·^−^ content	150.692	<0.001	4160.150	<0.001	43.367	<0.001
	Chlorophyll a content	319.466	<0.001	933.925	<0.001	35.483	<0.001
	Chlorophyll b content	205.963	<0.001	475.624	<0.001	6.492	<0.001
	Carotenoid content	195.133	<0.001	1381.246	<0.001	51.281	<0.001
	Soluble protein content	264.448	<0.001	5750.398	<0.001	39.162	<0.001
	Total soluble sugar content	103.246	<0.001	1411.071	<0.001	9.913	<0.001

See legend to Fig. 1 for an explanation of treatment.
